# Death from Nitrous Oxide Gas

**Published:** 1872-04

**Authors:** 


*Death from Nitrous Oxide Gas.*—Several weeks ago Mrs. O'Shaughnessy died in the office of a dental practitioner, Dr. Newbrough, in West Thirty-fourth street, New York city, while under the influence of nitrous oxide gas, administered for the purpose of having teeth extracted without pain.

It was at first published in the daily papers that the verdict of the coroner's jury was death from congestion of the lungs superinduced by the gas. A later account states that a large number of dentists and others were present at the inquest, after much testimony on the several properties of nitrous oxide, a verdict was given of death from asphyxia, and that the jury condemned the manner in which the gas is generally manufactured. and the careless manner in which it is administered. We have at this time no further particulars.

